# Esophagectomy after definitive chemoradiation in esophageal cancer: a safe therapeutic strategy

**DOI:** 10.1093/dote/doae059

**Published:** 2024-08-08

**Authors:** Eline G M van Geffen, Karen J Neelis, Hein Putter, Marije Slingerland, Wobbe O de Steur, Jolein van der Kraan, Aart J van der Molen, A Stijn L P Crobach, Henk H Hartgrink

**Affiliations:** Department of Surgical Oncology, Leiden University Medical Center, Leiden, The Netherlands; Department of Radiation Oncology, Leiden University Medical Center, Leiden, The Netherlands; Department of Medical Statistics, Leiden University Medical Center, Leiden, The Netherlands; Department of Medical Oncology, Leiden University Medical Center, Leiden, The Netherlands; Department of Surgical Oncology, Leiden University Medical Center, Leiden, The Netherlands; Department of Gastroenterology, Leiden University Medical Center, Leiden, The Netherlands; Department of Radiology, Leiden University Medical Center, Leiden, The Netherlands; Department of Pathology, Leiden University Medical Center, Leiden, The Netherlands; Department of Surgical Oncology, Leiden University Medical Center, Leiden, The Netherlands

**Keywords:** anastomotic leakage, chemoradiation, esophageal cancer surgery, esophagectomy, therapy, treatment

## Abstract

The standard treatment regimen for esophageal cancer is chemoradiation followed by esophagectomy. However, the use of neoadjuvant chemoradiotherapy damages the surrounding tissue, which potentially increases the risk of postoperative complications, including anastomotic leakage. The impact of definitive chemoradiotherapy (dCRT, 50.4 Gy radiotherapy) compared to the standard neoadjuvant scheme (nCRT, 41.4 Gy radiotherapy) prior to surgery on the incidence of anastomotic leakage remains poorly understood. To study this, all patients who received dCRT between 2011 and 2021 followed by esophagectomy were included. For each patient, two patients who received nCRT were selected as matched controls. Outcomes included postoperative anastomotic leakage, pulmonary and other complications, anastomotic stenosis, pulmonary and other postoperative complications (Clavien Dindo Classification ≥1), and overall survival. One hundred and eight patients were included with a median follow-up of 28 months. The time between neoadjuvant treatment and surgery was longer in the dCRT group compared to the nCRT group (65 vs. 48 days, *P* < 0.001). Postoperatively, significantly more patients in the dCRT group suffered from anastomotic leakage (11% vs. 1%, *P* = 0.04) and anastomotic stenosis (42% vs. 17%, *P* < 0.01). No differences were found for other complications or overall survival between both groups. In conclusion, preoperative dCRT is associated with a higher risk of anastomotic leakage and stenosis. These complications, however, can be treated effectively. Therefore, esophagectomy after dCRT is considered to be an appropriate treatment strategy in a selected patient group.

## INTRODUCTION

Esophageal cancer is an aggressive form of cancer, with an annually increasing mortality of over 500 000 cases worldwide.^1^ The introduction of neoadjuvant chemoradiotherapy (nCRT) led to a clear survival benefit compared to surgery alone, subsequently establishing a new standard of care. The current neoadjuvant treatment regimen, based on the CROSS trial, entails CRT (carboplatin AUC 2/paclitaxel 50 mg/m^2^ and 41.4 Gy radiotherapy) followed by surgical resection of the esophagus.^2^^,^^3^ The CROSS treatment regimen substantially increased median overall survival from 24 months to 49 months compared to surgery alone.^4^

However, it should be noted that radiotherapy elicits an inflammatory response, which in turn stimulates excessive collagen production, resulting in the development of fibrosis.^5^ When CRT is administered prior to surgery, it has the potential to complicate the esophagectomy and may negatively affect postoperative outcomes in terms of anastomotic leakage and infection.^4^^,^^6^^,^^7^ Increasing the dose of radiotherapy supposedly leads to more cellular damage, tissue necrosis, and less perfusion, and may increase the risk of adverse events.^8–12^

In contrast to the current standard of care using 41.4 Gy of radiotherapy, other prospective studies have utilized radiotherapy doses ranging up to 45.0 Gy as part of preoperative management with satisfactory results.^13^ In the absence of intended surgery, patients receive a radiation dosage of 50.4 Gy in combination with chemotherapy, or even more in dose-escalation studies, as a definitive treatment with curative intent. However, in this group solely treated with CRT, the tumor recurrence rates are high (40%–75%).^8^^,^^11^ From those patients treated without surgery, a subset has residual disease or recurrence and will be considered for additional surgical resection, with a possible higher risk of adverse events due to the fibrosis within the irradiated field.^5^

Currently available literature reports contradictory outcomes on the risk of postoperative adverse events after higher dosages of radiotherapy prior to esophagectomy. Therefore, our study aimed to investigate the impact of definitive CRT (dCRT, 50.4 Gy) compared to standard neoadjuvant CRT (nCRT, 41.4 Gy) on anastomotic leakage and stenosis, pulmonary and other postoperative complications.

## METHODS

A single-center, retrospective case–control study was conducted in the Leiden University Medical Centre (LUMC) in the Netherlands. The study obtained central approval by the medical ethics board of the LUMC on 9 March 2022. All patients who underwent esophageal resection after CRT therapy at the LUMC between January 2011 and December 2020 were collected and divided into two groups based on neoadjuvant treatment: dCRT or nCRT. dCRT was defined as 50.4 Gy radiation therapy in combination with weekly carboplatin/paclitaxel. Patients who underwent dCRT or nCRT treatment but did not make it to surgery were not collected and therefore not included in the analysis. The nCRT treatment regimen entailed 41.4 Gy of radiation therapy, also in combination with weekly carboplatin/paclitaxel. Patients were excluded if surgical procedure included removal of the larynx.

Each dCRT patient was individually matched to two control patients from the same hospital who had received nCRT treatment according to a propensity matching score.^14^ Matching was based on age, gender, year, and type of surgery (transhiatal, transthoracic, laparoscopic, or open), the American Society of Anaesthesiologists’ classification of physical health (ASA), and type of tumor (adenocarcinoma or squamous cell carcinoma), and the two nearest neighbors were selected as the nCRT controls for each dCRT subject. Other tumor types (e.g. adenosquamous and neuroendocrine carcinomas) were not present in the dCRT group and therefore not included. Baseline data and short-term and long-term outcomes were collected from electronical patient files.

### Surgical techniques and follow-up

All patients were discussed in a multidisciplinary team meeting during which the preferred treatment strategy was determined by a team consisting of medical oncologists, surgical oncologists, gastroenterologists, radiation oncologists, pathologists, and (nuclear) radiologists. The decision to offer dCRT could depend on patient characteristics, patient preference, or clinical T4a tumor stage. All selected patients underwent esophagectomy after CRT. The approach of the esophagectomy depended on tumor location; a transthoracic surgical approach was performed for tumors in the proximal two-third of the esophagus, whereas a transhiatal approach was preferred for tumor locations in the distal one-third. All patients underwent restoring the digestive tract by gastric tube reconstruction with a cervical anastomosis.

Postoperatively, all patients had a nasogastric tube, which was placed during surgery, and were fed through a feeding jejunostomy for the first 5 days. At Day 5, a fluoroscopic examination of the gastric tube reconstruction was performed to check for anastomotic leakage and adequate passage. If no anastomotic leakage was detected, the nasogastric tube was removed, and patients were allowed to expand dietary intake. Adjuvant therapy was not administered to any of the patients in the cohort. For the first year of follow-up, patients were monitored in the outpatient clinic every 3 months, during which anamnesis and physical examination were performed according to the Dutch guidelines.^15^ This frequency is reduced to every 6 months in the second year, and after that, the follow-up frequency is further reduced to once a year. Follow-up during the COVID pandemic took place through telephonic appointments. After a recurrence-free period of 5 years, patients were dismissed from routine follow-up.

### Outcomes

The primary outcome of this study was anastomotic leakage and corresponding CD grade. Anastomotic leakage was defined as leakage of (water-soluble) contrast medium at fluoroscopy or an anastomotic defect diagnosed during endoscopy, leading to adjustments in planned treatment (elongation of nasogastric tube dependency, antibiotic treatment, or surgery). Secondary outcomes included complications during hospitalization (general and pulmonary), anastomotic stenosis (including number of dilations), and overall survival. Anastomotic stenosis was endoscopically diagnosed and included if in need of anastomotic dilation. General complications included all deviations from the normal postoperative course and included wound infections, supplementary minerals, atrial fibrillation, and more. Examples of pulmonary complications include pulmonia, pneumothorax, and respiratory deficiency. All complications were classified according to the Clavien–Dindo classification (C-D classification).^16^ A complete pathological tumor response was defined as the absence of a tumor in the pathological specimen. Moreover, a sub-analysis to investigate the development of anastomotic stenosis after anastomotic leakage was made in participants diagnosed with anastomotic leakage.

### Statistical analyses

Statistical analysis was conducted in SPSS version 28. Means with standard deviations were calculated for normally distributed data, and medians with an interquartile range were calculated for not normally distributed data. Independent *T*-tests, Fisher’s exact test, and X^2^ tests were used to compare between groups, depending on the distribution of the data. A Kaplan–Meier survival analysis was used to generate survival data; a log-rank test was used to compare survival data between the groups. Considering the limited number of patients who underwent dCRT, a multivariate analysis could not be conducted. Statistical significance was set at a *P*-value of 0.05.

## RESULTS

Between January 2011 and December 2020, 511 patients underwent an esophagectomy for esophageal cancer in the LUMC. Thirty-six (7%) of all patients received dCRT before esophagectomy. For 23 patients (64%), the indication for dCRT stemmed from uncertainties regarding the resectability of the tumor, while for 7 patients (19%), the decision was driven by the presence of comorbidities or the patients’ medical condition, necessitating dCRT to allow for extended prehabilitation. In three cases (8%), tumor regrowth occurred following a period of active surveillance, prompting the consideration of salvage esophagectomy. Additionally, in two cases (6%), patients opted for dCRT due to ambivalence toward surgical intervention. Notably, one patient was offered dCRT due to prolonged surgical waiting times resulting from the COVID-19 pandemic. After matching each dCRT patient to two patients with nCRT, 108 patients were included.

Table 1 provides demographical and clinical characteristics of the included patients. The average age of the cohort was 67 years (SD 7) and 70% was male. The tumor type, type of surgery, blood loss during surgery, and duration of the surgery were equally distributed between the groups. One of the included patients had synchronous liver metastases. This patient was initially treated with chemotherapy and received dCRT followed by an esophagectomy after being free from metastases for 2 years but with a residual disease in the esophagus. The time between CRT and surgery was significantly longer for the dCRT group as compared to nCRT (65 days [IQR 51–97] vs. 48 days [IQR 41–54], *P* < 0.001). Median follow-up after surgery was 29 months (IQR 12–50), and the average postoperative in hospital stay was 8 days.

**Table 1:** Baseline characteristics

	**dCRT *n* = 36, (%)**	**nCRT *n* = 72, (%)**	***P*-value**
**Gender:** male	27 (75)	49 (68)	0.5
**Age** (mean, SD)	67.9 (7.6)	66.4 (7.3)	0.3
**ASA**			0.2
I	3 (8)	4 (6)	
II	21 (58)	53 (74)	
III	12 (33)	15 (21)	
**cT-stage**			<0.01
2	5 (14)	16 (22)	
3	23 (64)	55 (76)	
4	8 (22)	1 (1)	
**cN-stage**			0.84
0	13 (36)	26 (36)	
1	12 (33)	38 (39)	
2	10 (28)	15 (21)	
3	1 (3)	3 (4)	
**cM-stage**			0.33
0	35 (97)	72 (100)	
1	1 (3)	0 (0)	
**Tumor type:**			>0.90
Adenocarcinoma	17 (47)	34 (47)	
Squamous cell carcinoma	19 (53)	38 (53)	
**Days from CRT to surgery** (median, range)	64.5 (51–97)	48.0 (41–54)	<0.01
**Type of surgery**			0.8
Transhiatal	18 (50)	36 (50)	
Transthoracal (open)	13 (36)	29 (40)	
Transthoracal (laparoscopic)	5 (14)	7 (10)	
**Year of surgery**			>0.90
2012–2014	8 (22)	16 (22)	
2015–2017	20 (56)	40 (56)	
2018–2020	8 (22)	16 (22)	
**Surgery time** (minutes, median, range)	223 (100–360)	216 (120–440)	0.5
**Blood loss** (ml, median, range)	450 (95–1400)	350 (30–1900)	0.09

### Postoperative complications

Table 2 shows the complications, including anastomotic leakage, pulmonary complications, and other complications with corresponding complication grade. In the group that received dCRT, significantly more people suffered from postoperative anastomotic leakage compared to the nCRT group (11% vs. 1%, *P* = 0.04). The corresponding complication grades were 2 or 3 based on the C&D classification and included a mediastinal infection, recovery by additional surgery, or recovery by an anastomotic stent or antibiotic in the dCRT group, whereas the case that suffered from anastomotic leakage in the nCRT group was classified as grade 1 according to the C&D classification and treated with an elongation of the nasogastric tube period and prolonged parenteral feeding.

**Table 2:** Short term complications

	**dCRT *n* = 36, (%)**	**nCRT *n* = 72, (%)**	***P*-value**
**Anastomotic leakage**	4 (11)	1 (1)	0.04
CDC 1	0 (0)	1 (100)	
CDC 2	1 (25)	0 (0)	
CDC 3a	1 (25)	0 (0)	
CDC 3b	2 (50)	0 (0)	
**Pulmonary complications**	12 (33)	22 (31)	0.8
CDC 1	2 (17)	4 (18)	
CDC 2	6 (50)	12 (55)	
CDC 3a	1 (8)	3 (14)	
CDC 3b	1 (8)	2 (9)	
CDC 4	1 (8)	1 (5)	
CDC 5	1 (8)	0 (0)	
**Other complications**	20 (56)	31 (43)	0.23
CDC 1	6 (29)	5 (16)	
CDC 2	12 (57)	21 (68)	
CDC 3a	0	2 (6)	
CDC 3b	1 (5)	3 (10)	
CDC 4	0	0	
CDC 5	1 (5)	0	
**Anastomotic stenosis**	15 (42)	12 (17)	<0.01

Pulmonary and other complications did not differ between the nCRT and the dCRT group (33% vs. 31% and 55% vs. 43%, respectively, Table 2). During the follow up, significantly more patients developed anastomotic stenosis in the dCRT group compared to the nCRT group (36% vs. 17%, *P* = 0.02). The time from surgery to diagnosis of anastomotic stenosis was not statistically different in the nCRT (median 167 days, IQR 86–167) compared to the dCRT cohort (median 88 days, IQR 64–268, *P* = 0.304). In the dCRT group, on average, 8 dilations were performed during follow-up, compared to 5 in the nCRT group (*P* = 0.12). Additional analysis of whether anastomotic stenosis occurred more in the anastomotic leakage group showed only one case (20%) in the anastomotic leakage group (*n* = 5), compared to 26 cases (25%) in the patients without leakage (*n* = 103, *P* > 0.90).

### Survival

**Fig. 1:**
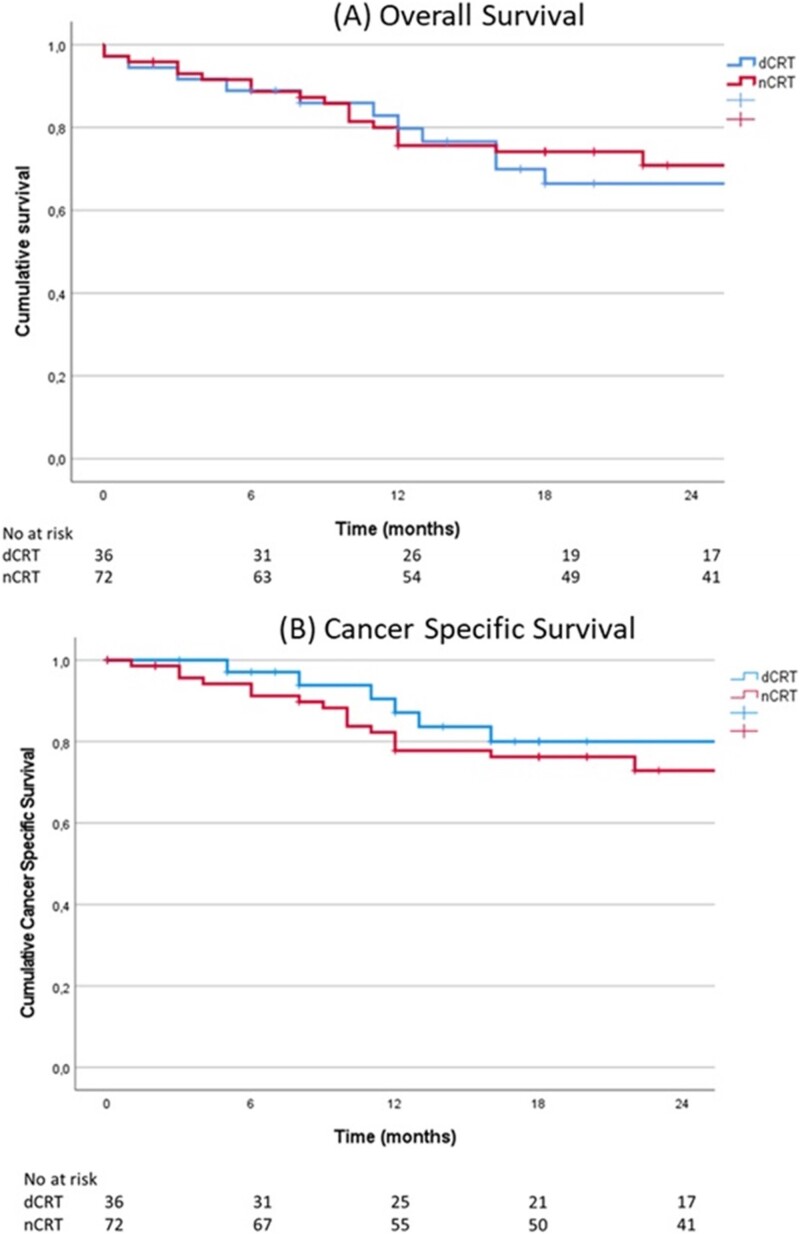
Survival analysis: (Abbreviations: (definitive/neoadjuvant) chemoradiotherapy [(d/n)CRT]). (A) Two-year overall survival for patients with nCRT (70%) and patients with dCRT (66%, *P* = 0.67). (B) Two-year cancer-specific survival for patients with nCRT (73%) and patients with dCRT (80%, *P* = 0.41).

As shown in Fig. 1, the overall and cancer-specific survival between both groups was similar during the median follow-up period of 2 years. After 2 years, 66% of the dCRT group was still alive, compared to 70% in the nCRT group (*P* = 0.67). Two patients were deceased during postoperative hospitalization, one because of a massive hemorrhagic stroke and one because of pulmonary complications, both in the dCRT group.

### Tumor response

In the dCRT group, 14 patients (39%) had a complete pathological response, and 22 patients (61%) had a residual tumor in the resection specimen. In the nCRT group, 23 patients had a complete response (32%), while 49 patients had a partial or no response (68%, *P* = 0.50). Patients in the dCRT cohort had a ypT0-stage in 42% (*n* = 15), compared to 38% (*n* = 27) in the nCRT cohort (*P* = 0.68). Pathological response for ypN-stage was also comparable for both cohorts (*P* = 0.81): after dCRT 47% achieved pCR (compared to 46% after nCRT), 17% did not achieve pCR (compared to 18% after nCRT), 28% had a cN0 and ypN0 stage (compared to 22% after nCRT), and 8% had pathological lymph nodes in the resection specimen but not on pre-operative imaging (compared to 14% after nCRT).

## DISCUSSION

This retrospective study examined a cohort of 36 patients who underwent dCRT followed by esophagectomy for esophageal cancer at the LUMC. This study aimed to compare the incidence of adverse events and survival outcomes with a control group of 72 matched patients who received standard nCRT followed by esophagectomy. The findings of this study suggest that administration of higher doses of radiotherapy (50.4 Gy vs. 41.4 Gy) was associated with an increased risk of anastomotic leakage, with an incidence of 11% in the dCRT group compared to 1% in the nCRT group and to increase the likelihood of developing anastomotic stenosis to 14% (vs. 4%). These results may indicate a potential dose-dependent relationship between radiation dose and the occurrence of these adverse events.

Several previous studies have reported contradicting outcomes regarding the impact of dCRT prior to esophagectomy on postoperative events. Haque et al. conducted a study using the American National Cancer Data Base, selecting 257 cases with high dosage RT (50.0–50.4 Gy) and comparing them with 4768 patients receiving low dosage RT (40.0–41.4 Gy), resulting in similar complication rates.^17^ Another study by Jamel et al. conducted a review involving 563 patients receiving dCRT in combination with surgery compared to 1343 patients receiving nCRT and reported a doubled incidence of anastomotic leakage and increased postoperative morbidity for those who had received dCRT.^18^ Although these studies do not provide details regarding the used radiosensitizer, it is worth noting that cisplatin and capecitabine are known to be associated with more toxicity compared to carboplatin and taxol.^19^ Nevertheless, the study by Jamel et al. and the current study both support the notion that higher doses of radiation therapy have a negative impact on postoperative outcomes.

In the current study, anastomotic leakage led to severe adverse events in the dCRT group, ranging from mediastinal infection to the need for additional surgery, anastomotic stents or antibiotics, and extended treatment with a nasogastric tube. Although information on radiation fields in both groups was not analyzed, the extent of these fields might have affected these outcomes. Nevertheless, all four patients in the dCRT group with anastomotic leakage were successfully treated for their anastomotic leakage, and patients with stenosis were successfully treated with esophageal dilatation. Despite this, anastomotic leakage and stenosis might have an impact on quality of life. Unfortunately, for this cohort, no information regarding quality of life was available. However, in light of these complications, it is important to carefully inform the patients who selected for dCRT of the risk of these complications before performing an esophagectomy.

Analysis of the short-term complications in this study revealed comparable postoperative complications between the nCRT and dCRT groups, other than anastomotic leakage and stenosis. However, since only two treatment regimens were compared, it was not possible to determine a specific cut-off dosage of radiotherapy after which the complication rate increased. This study also found a similar 2-year overall survival for the nCRT and dCRT groups (70% and 66%, respectively). This study did not analyze dCRT alone compared to dCRT followed by esophagectomy, and it can be expected that this study population differs due to a selection bias favoring those fit for surgery. Nevertheless, the 2-year overall survival seems higher compared to previous studies analyzing dCRT alone, which report a 3-year overall survival of 42%. With these limitations in mind, these findings suggest that dCRT followed by esophagectomy could be an oncological safe strategy with similar overall survival to patients with nCRT followed by surgical resection.

The previously published ART-DECO trial reports that higher radiation doses do not seem to improve local control.^11^ Moreover, the soon expected SANO-trial will shred a new light on active surveillance for those with a complete response, thereby avoiding the risk of anastomotic leakage or stenosis.^20^ Additionally, part of the patients in the SANO-trial are expected to undergo delayed surgery, which may add valuable information concerning the effect of the increased time-interval on the studied outcomes. There is a possibility that not only dCRT but also the increased time between neoadjuvant treatment and surgery might contribute to the development of fibrosis in the surgical plane, resulting in more difficult dissection and thereby increasing the anatomic leakage and stenosis rate.

A new development in esophageal cancer treatment involves adjuvant immunotherapy (nivolumab) for patients with locally advanced esophageal cancer who have residual tumor in the resection specimen after nCRT and surgical resection.^21^ Adjuvant nivolumab after CRT and surgical resection has shown a clear survival benefit compared to placebo in locally-advanced esophageal cancer.^21^ However, in this trial, the impact of adjuvant nivolumab has not been adjusted for varying neoadjuvant dosages of CRT. Future studies are needed to investigate whether nCRT and dCRT patients derive similar benefits from adjuvant nivolumab.

It is important to consider the limitations of this study when interpretating the obtained results. First, data were extracted for a limited number of included patients from one center, in which esophagectomies are performed by two surgeons. Consequently, the generalizability of the findings may be limited. The LUMC is a specialized center, performing approximately 50 esophagectomies per year with an average anastomotic leakage rate of 4.3%.^22^ While cervical anastomosis is associated with a higher anastomotic leakage rate,^23^ the LUMC exclusively performs cervical anastomosis with good results. Moreover, selection of patients from a surgical database restricts the ability to contextualize these results in relation to the proportion of individuals receiving a CRT regimen not followed by surgery. Furthermore, patients with dCRT followed by esophagectomy are scarce, resulting in a limited sample-size of only 36 patients out of a total of 511 esophagectomies performed in the 10-year inclusion period. This fact, combined with the retrospective nature of the study and matching of patients, induces the possibility of selection bias affecting the outcomes. Patients were matched with controls based on ASA classification, along with age, gender, year, and type of surgery, since, e.g. ASA classification has been previously shown to negatively influence the chance of developing anastomotic leakage.^23^^,^^24^ The higher cT-stage at baseline might have influenced outcomes; however, cT-stage is not known to influence anastomotic leakage rate and was therefore not included in the matching process. One patient with a M1 status was included, because a favorable oncological response was found after chemotherapy. This patient was not removed in the matching process because this was not thought to affect the anastomotic leakage of stenosis rate. Besides cT and cM-stage, the groups were similar at baseline, indicating that the selection of the control group was executed effectively.

Retrospectively including patients who underwent dCRT introduces inherent differences compared to those who receive nCRT, as various reasons can influence the choice of treatment and might have contributed to the delay to surgery in the dCRT group. First of all, tumors that were initially deemed irresectable, might become resectable due to tumor shrinkage during treatment, leading to subsequent esophagectomy. Additionally, patients who initially declined surgery may change their preference and opt for surgical resection after dCRT treatment. A third group includes patients who have a recurrence of the tumor after dCRT, and therefore, salvage esophagectomy is indicated. The last group consists of people who were initially unfit for surgery, or had multi-morbidities requiring urgent treatment, but were re-evaluated and were considered fit for surgery after dCRT. These reasons have probably contributed to a significant increase for the dCRT group in time between the CRT and surgical treatment CRT from 48 days in nCRT to 65 days in dCRT (*P* < 0.01). While this study outlines the rationale for dCRT in the respective patient group, it is essential to recognize the intricate nature of multidisciplinary decision-making, which is patient-tailored. Consequently, no conclusions can be drawn regarding whether the potential risk of anastomotic leakages and stenosis could have been spared.

This study offers insight into the postoperative complications after dCRT followed by esophagectomy, leading to an increased incidence of anastomotic problems (leakage and stenosis). The results of this study therefore indicate that patients should be carefully selected when esophagectomy after dCRT is planned and should be informed of the increased risks of complications. A prospective national cohort study could be useful to identify and characterize this group in order to make more specific guidelines for patient selection to avoid complications after surgery. A study considering the quality of life of esophageal cancer patients is currently being conducted in The Netherlands and may give insight into the consequences of the dCRT and nCRT regimens and the impact of increased anastomotic leakage and stenosis rates.^25^

## CONCLUSION

Esophagectomy after dCRT is associated with a higher anastomotic leakage and anastomotic stenosis rate. These complications, however, can be treated effectively. Therefore, dCRT prior to surgery is considered an appropriate treatment strategy in a carefully selected group of patients.
